# Residence-based inequalities in health facility delivery among women in sub-Saharan Africa

**DOI:** 10.1093/inthealth/ihag030

**Published:** 2026-03-26

**Authors:** Richard Gyan Aboagye, Joshua Okyere, Precious Adade Duodu, Florence Gyembuzie Wongnaah, Bright Opoku Ahinkorah

**Affiliations:** Department of Family and Community Health, Fred N. Binka School of Public Health, University of Health and Allied Sciences, Hohoe, Ghana; School of Population Health, University of New South Wales, Sydney, NSW 2052, Australia; School of Human and Health Sciences, University of Huddersfield, Queensgate, Huddersfield, England, UK; College of Health Sciences, University of Ghana, Accra, Ghana; Department of Nursing, School of Human and Health Sciences, University of Huddersfield, Queensgate, Huddersfield, UK; Department of Nursing, School of Nursing and Midwifery, College of Health Sciences, Kwame Nkrumah University of Science and Technology (KNUST), Ghana; Department of Global Public Health, Karolinska Institutet, Stockholm, Sweden; REMS Consultancy Services, Sekondi-Takoradi, Western Region, Ghana; College of Health, Adelaide University, Adelaide, SA, Australia

**Keywords:** Demographic and Health Survey, health facility delivery, inequality, place of delivery, place of residence, sub-Saharan Africa

## Abstract

**Background:**

Access to skilled healthcare during childbirth is essential, yet disparities persist, particularly in rural areas in sub-Saharan Africa. This study examines the factors associated with rural-urban disparities in health facility delivery in sub-Saharan Africa.

**Methods:**

We utilised data from the Demographic and Health Surveys conducted in 23 sub-Saharan African countries from 2015 to 2022. We employed a multivariate non-linear decomposition model to examine the factors contributing to rural–urban disparities in health facility delivery. This method divides differences in mean values between two groups into components attributed to observed characteristics and their estimated effects using a regression model. Results were categorised into differences due to attributes and coefficients, with each group presenting coefficients, 95% confidence intervals, p-values and percentages.

**Results:**

The decomposition analysis revealed that 87.13% of the observed rural-urban disparity in health facility delivery was explained by differences in women’s characteristics. The most significant contributors were in the richest wealth index (15.62%) and having four or more antenatal care visits (10.55%). Other notable factors included internet use (4.72%) and lack of formal education (4.43%).

**Conclusions:**

This study identified significant rural–urban disparities in health facility delivery in sub-Saharan Africa, favouring urban areas. Key contributing factors included wealth status, antenatal care visits, internet use and educational attainment. Improving health facility delivery in rural areas requires enhancing women’s economic status, encouraging more antenatal care visits and prioritising interventions for women without formal education.

## Introduction

Maternal mortality and under-five mortality, including neonatal mortality, remain significant global health concerns. Maternal mortality refers to ‘death of a woman during pregnancy or within 42 days of termination of a pregnancy, irrespective of the duration and site of the pregnancy, from any cause related to or aggravated by the pregnancy or its management, but not from accidental or incidental causes’.^1^ Alternatively, neonatal death is defined as ‘deaths during the first 28 completed days of life per 1000 live births in a given year or other period’.^2^ In 2020, one woman died every 2 minutes, with approximately 800 dying daily due to preventable pregnancy and childbirth-related causes.^3^ About 75% of these global maternal deaths resulted from severe haemorrhage or bleeding, infections, pre-eclampsia and eclampsia, complications during childbirth and unsafe abortion.^3,4^

In 2020, an alarming 95% of all maternal deaths occurred in developing countries, particularly sub-Saharan Africa (SSA).^3^ This has mainly been attributed to inadequate healthcare facilities and limited access to healthcare services. Additionally, pregnant women often do not utilise available health facilities for delivery due to religious beliefs, cultural customs, or financial constraints to access healthcare services.^5,6^ Substantial progress has been made over the past 2 decades (2000–2020), resulting in a 34.2% reduction in the global maternal mortality ratio (MMR) and a 33% decrease in the MMR in SSA.^3^ While this positive trend is encouraging, it conceals significant intra- and intercountry inequities across the African region. Unsurprisingly, maternal mortality and morbidity remain unacceptably high and prevalent in Africa, with the region accounting for about two-thirds (70%) of global maternal deaths, which represented 531–536 deaths per 100 000 live births in 2020.^7^ Furthermore, SSA experienced a considerable disparity, contributing to 57% (2.8 [range 2.5–3.3] million) of the total under-five deaths globally, despite SSA making up only 30% of global live births. Additionally, SSA reported the highest neonatal mortality rate globally, reaching 27 deaths per 1000 live births.^8^

A critical determinant of maternal health outcomes is the place of delivery. Achieving safe, high-quality and skilled maternal healthcare by health professionals at the right time (during pregnancy, at childbirth and postpartum) is essential for significantly reducing maternal and neonatal mortalities and enhancing health outcomes.^3^ Health facility deliveries are associated with lower risks of complications and adverse maternal and neonatal outcomes,^9,10^ although this does not necessarily imply a survival benefit for women or babies in some cases,^11^ particularly those with low facility capability^12^ and provider skill.^13^

According to the World Health Organization (WHO) and the United Nations Children’s Fund,^14^ the proportions of births attended by skilled healthcare professionals such as midwives, doctors or nurses are lower in low-income (68%) and lower-middle-income countries (78%), including those in SSA, compared with most high-income and upper-middle-income countries (99%). Consequently, disparities in the utilisation of health facility delivery services persist, especially in SSA, where a significant number of women continue to give birth at home, often without access to skilled birth attendants.^15–17^ Therefore, it is crucial to understand the factors contributing to these disparities, particularly the impact of place of residence (urban or rural) on maternal healthcare utilisation. In SSA, where most of the population lives in rural areas and faces socio-economic challenges, it is vital to investigate how residence status affects utilisation of health facilities. Differences between rural and urban settings in access to healthcare infrastructure, information and socio-economic resources can lead to pronounced disparities in health facility delivery rates.^18^

While several studies have examined maternal healthcare utilisation, including health facility delivery, using data from the Demographic and Health Surveys (DHS),^19,20^ limited research has employed decomposition analysis to quantify the specific contributions of residence-based disparities to inequalities in health facility delivery across SSA. Therefore, this study examines the influence of residence status on facility-based delivery among women in SSA. It employs decomposition analysis to disaggregate the role of residential disparities. This approach enhances the understanding of the factors driving inequalities, highlighting their respective contributions to overall disparities in health facility delivery. By doing so, the study aims to provide a nuanced understanding of the factors influencing maternal healthcare utilisation in the region and to offer evidence-based recommendations for more equitable and effective interventions. Ultimately, the findings will contribute to developing targeted interventions to achieve the Sustainable Development Goals (SDGs), with target 1 of SDG 3 aiming to ‘reduce the global maternal mortality rate to less than 70 deaths per 100 000 live births by 2030’.^3,21^ It has been reported that a yearly reduction of at least 11.6% and 20.3% in maternal mortality rate from 2020 is required at the global and African regional levels, respectively, to meet this target.^3,14^

## Methods

### Data source and design

The study utilised data from the DHS conducted in 23 sub-Saharan African countries. These surveys were carried out from 2015 to 2022 and the list of these countries can be found in Table 1. The DHS is a representative survey that aims to enhance understanding of health and demographic patterns in low- and middle-income countries (LMICs).^22^ Since its establishment in 1984, the DHS has conducted >350 surveys in >90 countries.^22^ A cross-sectional design was employed for the DHS and a multistage cluster sampling method was used to select respondents. Data were collected using a pretested structured questionnaire. The detailed methodology of the DHS has been discussed in the literature.^23^ The questionnaire covered various health, socio-economic and demographic topics, including health facility delivery.^22^ The study included 172 793 women of reproductive age (15–49 years) who had previously given birth before the DHS was conducted. The sample sizes for each country are shown in Table 1. The study adhered to the Strengthening the Reporting of Observational Studies in Epidemiology (STROBE) guidelines for reporting observational studies.^24^

**Table 1.** Description of study sample.

Countries	Year of survey	Weighted sample (n)	Weighted percentage
Angola	2015–2016	6566	3.80
Burkina Faso	2021	8407	4.87
Benin	2017–2018	7692	4.45
Burundi	2016–2017	8575	4.96
Cameroon	2018	7222	4.18
Côte d’Ivoire	2021	6647	3.85
Ethiopia	2016	7959	4.61
Gabon	2019–2021	5180	3.00
Gambia	2019–2020	5286	3.06
Guinea	2018	5190	3.00
Liberia	2019–2020	3660	2.12
Madagascar	2021	8994	5.21
Mali	2018	5262	3.05
Mauritania	2019–2021	7622	4.41
Malawi	2015–2016	11 873	6.87
Nigeria	2018	20 224	11.70
Rwanda	2019–2020	7192	4.16
Sierra Leone	2019	7439	4.31
South Africa	2016	4139	2.40
Tanzania	2022	7379	4.27
Uganda	2016	8805	5.10
Zambia	2018	6539	3.78
Zimbabwe	2015	4941	2.86
All countries	2015–2022	172 793	100.00

### Variables

#### Outcome variable

The outcome variable in this study was health facility delivery, which was assessed by asking women, ‘Where did you give birth to [name of child]?’ The response options included hospitals, clinics, health centres, polyclinics, maternity homes, health posts and community-based health planning and services (CHPS), among others. These facilities were categorised as government-owned, private or non-governmental organisation owned. Women who delivered their babies in any of these facilities were recorded as 1 (yes) for ‘delivered in a health facility’, while those who did not deliver in a health facility were recorded as 0 (no).^25–31^

#### Stratifier

The type of residence was used as an indicator of inequality. Respondents were asked to indicate whether they lived in an urban or rural area, with 1 and 2 representing urban and rural, respectively. In the final analysis, we recoded the responses so that 1=0 represented urban and 2=1 represented rural.

#### Explanatory variables

Eighteen explanatory variables were considered for inclusion in the study. We selected these variables based on their association with health facility delivery or place of delivery from the literature^25–31^ and their availability in the DHS dataset. The variables consisted of the age of the woman, current working status, educational attainment, marital status, parity, age at first birth, health insurance coverage, antenatal care (ANC) visits, exposure to television, exposure to radio, exposure to reading newspapers or magazines, internet usage, wealth index, sex of household head as well as problems in women assessing healthcare for themselves categorized by the type of problem: difficulty in obtaining permission to seek medical care, financial constraints in obtaining medical treatment, long distance to a health facility as a barrier to care and absence of someone to accompany them to the health facility.

### Statistical analyses

All analyses were conducted using Stata version 17.0 (StataCorp, College Station, TX, USA). We used the ‘spmap’ function in Stata to display the proportion of women who delivered in a health facility. We then estimated the proportion of health facility deliveries for each rural–urban stratum, along with their associated 95% confidence intervals (CIs). Covariates included in the regression models were selected based on a combination of theoretical relevance, prior empirical evidence and statistical model fit and parsimony. We initially considered variables known to influence health facility delivery in SSA. To refine the model, we applied the ‘gvselect’ command in Stata and compared candidate models using the Akaike information criterion (AIC), selecting the model with the lowest AIC (i.e. the best-fitting model). This approach ensured that covariates were both epidemiologically meaningful and statistically supported.^32,33^ We used multivariable binary logistic regression to examine factors associated with health facility delivery within each rural–urban stratum. The results were presented as adjusted odds ratios (aORs) with their respective 95% CIs. To examine the factors contributing to rural–urban differences in health facility delivery, we used Powers et al.’s^34^ multivariate non-linear decomposition model with the Stata command ‘mvdcmp’. According to Powers et al.^34^ decomposition analysis partitions a difference in mean values between two groups into components resulting from group differences in observed characteristics and in the estimated effects of those characteristics using a regression model. The results were divided into two categories: differences due to attributes and differences due to coefficients. Each group displayed the coefficients, 95% CIs, p-values and percentages.

## Results

### Background characteristics of the respondents

#### Urban

Most women were 25–29 years of age (27.3%) in urban areas, had an incomplete secondary education (31.9%), were currently employed (58.4%), were married (60.3%) and experienced multiparity (53.0%). The age at first birth was evenly split, with 49.6% giving birth during adolescence. Majority (69.7%) attended ANC at least four times. More than half of the women had no health insurance subscription (86.8%), whereas 75.2% did not view distance to the health facility as a problem. Most urban women reported watching television (71.5%) and not using the internet (63.8%), as well as belonging to the richest wealth quintile (40.4%).

#### Rural

Most rural residents were 25–29 years of age (24.2%), had no formal education (43.2%), were currently employed (65.0%), were married (71.5%) and experienced multiparity (46.8%). First births mainly took place during adolescence, accounting for 62.3%. Fewer rural women (52.4%) attended four or more ANC visits. A larger proportion (92.4%) lacked health insurance and faced greater healthcare access challenges, with 46.5% considering distance a big problem. Most rural women did not watch television (76.8%) or use the internet (94.9%) and belonged to the poorest wealth quintile (30.8%) (see Table 2).

**Table 2.** Background characteristics of the women in SSA.

Variables	Urban (N=56 596), n (%)	Rural (N=116 197), n (%)
Woman’s age (years)		
15–19	3562 (6.3)	9334 (8.0)
20–24	12 180 (21.5)	26 628 (22.9)
25–29	15 434 (27.3)	28 123 (24.2)
30–34	12 239 (21.6)	22 969 (19.8)
35–39	8558 (15.1)	17 175 (14.8)
40–44	3690 (6.5)	8836 (7.6)
45–49	933 (1.7)	3131 (2.7)
Educational attainment		
None	12 266 (21.7)	50 613 (43.6)
Incomplete primary	7935 (14.0)	31 032 (26.7)
Complete primary	5207 (9.2)	12 726 (10.9)
Incomplete secondary	18 043 (31.9)	16 534 (14.2)
Complete secondary	7519 (13.3)	3900 (3.4)
Higher	5626 (9.9)	1393 (1.2)
Current working status		
Not working	23 542 (41.6)	40 699 (35.0)
Working	33 054 (58.4)	75 498 (65.0)
Marital status		
Never	7644 (13.5)	6931 (6.0)
Married	34 113 (60.3)	83 120 (71.5)
Cohabiting	10 680 (18.9)	17 887 (15.4)
Widowed	633 (1.1)	1527 (1.3)
Divorced	1246 (2.2)	2430 (2.1)
Separated	2280 (4.0)	4302 (3.7)
Parity		
Primiparity	14 746 (26.1)	22 548 (19.4)
Multiparity	29 994 (53.0)	54 376 (46.8)
Grandparity	11 856 (20.9)	39 273 (33.8)
Age at first birth		
Adult	28 540 (50.4)	43 802 (37.7)
Adolescent	28 056 (49.6)	72 395 (62.3)
Number of ANC visits		
0	4313 (7.6)	16 192 (13.9)
1–3	12 848 (22.7)	39 134 (33.7)
≥4	39 435 (69.7)	60 871 (52.4)
Covered by health insurance		
No	49 129 (86.8)	107 379 (92.4)
Yes	7467 (13.2)	8818 (7.6)
Watches television		
No	16 149 (28.5)	89 296 (76.8)
Yes	40 447 (71.5)	26 901 (23.2)
Listens to radio		
No	21 867 (38.6)	61 270 (52.7)
Yes	34 729 (61.4)	54 927 (47.3)
Reads newspaper or magazine		
No	42 800 (75.6)	105 737 (91.0)
Yes	13 796 (24.4)	10 460 (9.0)
Use internet		
No	36 136 (63.8)	110 251 (94.9)
Yes	20 460 (36.2)	5946 (5.1)
Getting medical help for self		
Permission to go		
Not a big problem	46 915 (82.9)	93 627 (80.6)
Big problem	9681 (17.1)	22 570 (19.4)
Distance to health facility		
Not a big problem	42 579 (75.2)	62 147 (53.5)
Big problem	14 017 (24.8)	54 050 (46.5)
Getting money for treatment		
Not a big problem	31 549 (55.7)	48 558 (41.8)
Big problem	25 047 (44.3)	67 639 (58.2)
Not wanting to go alone		
Not a big problem	47 986 (84.8)	85 206 (73.3)
Big problem	8610 (15.2)	30 991 (26.7)
Sex of household head		
Male	41 876 (74.0)	93 618 (80.6)
Female	14 720 (26.0)	22 579 (19.4)
Wealth index		
Poorest	2735 (4.8)	35 784 (30.8)
Poorer	4824 (8.5)	32 621 (28.1)
Middle	9235 (16.3)	25 701 (22.1)
Richer	16 970 (30.0)	16 018 (13.8)
Richest	22 832 (40.4)	6073 (5.2)

### Prevalence of health facility delivery among women in SSA

Figure 1 shows the prevalence of health facility delivery among women in SSA. Burkina Faso (98.2%), Rwanda (97.8%), South Africa (97.6%), Gabon (96.7%) and Malawi (96.5%) reported the highest health facility deliveries. In urban areas, the countries with the highest proportions of health facility delivery were South Africa (93.4%), Burkina Faso (92.6%), Rwanda (92.4%), Malawi (91.6%), Burundi (83.2%) and Sierra Leone (81.6%). Most of the deliveries were reported in urban areas (85.9%) compared with rural areas (64.7%) (Table 3).

**Figure 1.**
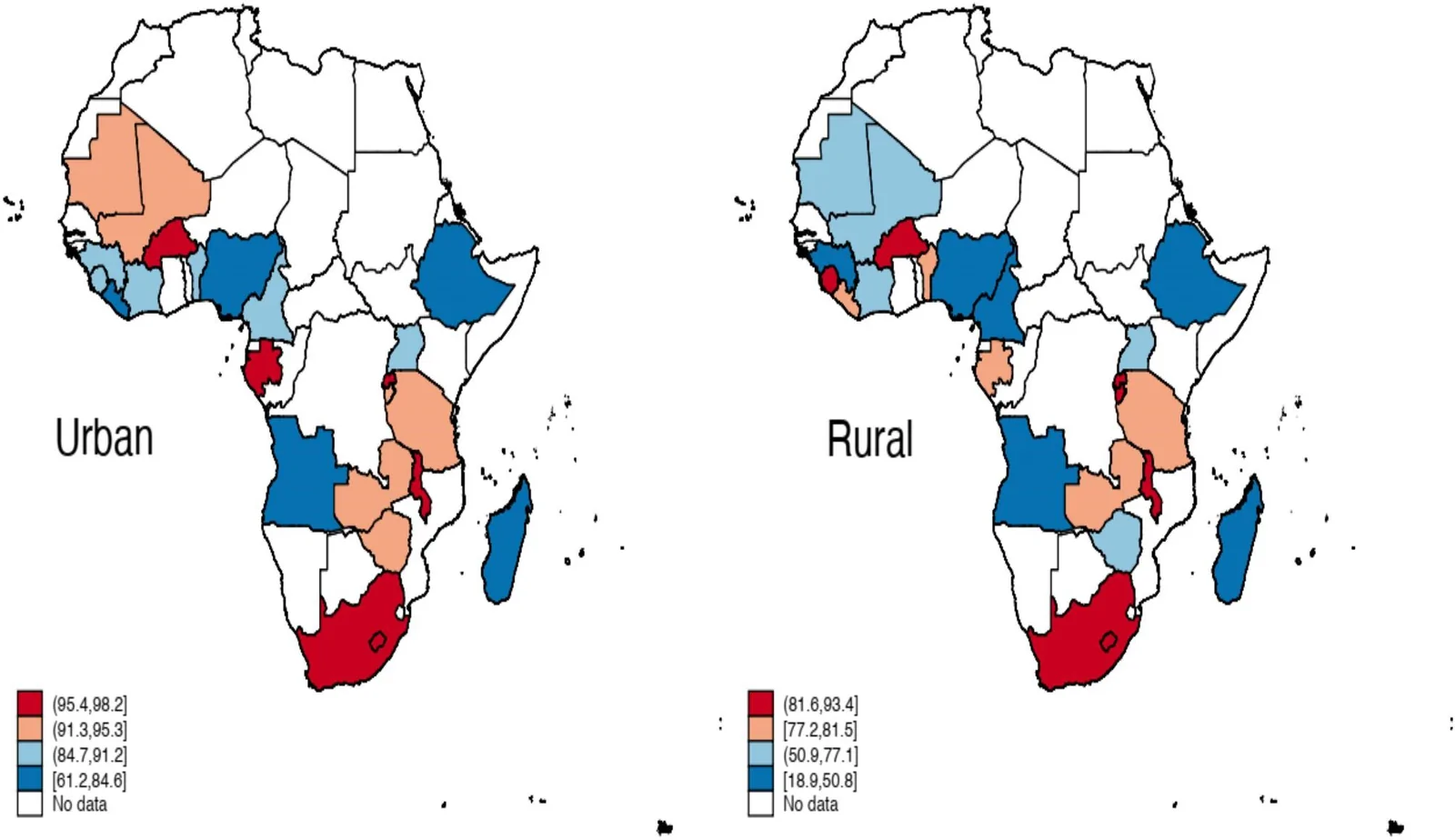
Prevalence of health facility delivery in urban and rural SSA.

### Bivariable analysis of health facility delivery among women in SSA per place of residence

Table 3 shows the prevalence of health facility delivery across the explanatory variables by place of residence. Among urban women, the prevalence of women who delivered at the health facility was high among those ages 30–34 years (87.1%), those with higher education (95.4%), those not currently working (87.7%), those who have never been married (89.6%), primiparous women (90.8%), those who gave birth as adults (89.9%), those in the richest wealth quintile (92.7%) and those who had four or more ANC visits (89.4%). Also, women who had health insurance (96.2%), used the internet (94.3%), watched television (89.6%), listened to the radio (87.5%) and read newspapers or magazines (93.4%) reported a higher prevalence of health facility delivery. Women who viewed permission to go (86.2%), distance to the health facility (86.8%), getting money for treatment (87.9%) and not wanting to go alone (86.2%) as not a big problem reported higher health facility delivery.

**Table 3.** Bivariable analysis of health facility delivery among women in SSA per place of residence.

	Urban (85.9%)		Rural (64.7%)	
Characteristics	% (95% CI)	p-Value^a^	% (95% CI)	p-Value^a^
Woman’s age (years)		<0.001		<0.001
15–19	84.5 (82.7 to 86.2)		65.7 (64.2 to 67.2)	
20–24	85.8 (84.7 to 86.8)		68.0 (66.9 to 69.2)	
25–29	86.5 (85.5 to 87.3)		64.4 (63.2 to 65.5)	
30–34	87.1 (86.1 to 88.0)		64.9 (63.7 to 66.1)	
35–39	86.3 (85.2 to 87.4)		63.4 (62.2 to 64.6)	
40–44	82.8 (80.9 to 84.5)		60.0 (58.5 to 61.4)	
45–49	76.7 (73.1 to 79.9)		54.6 (52.3 to 56.9)	
Educational attainment		<0.001		<0.001
None	76.2 (74.3 to 78.0)		52.2 (50.8 to 53.6)	
Incomplete primary	82.0 (80.5 to 83.4)		69.1 (68.0 to 70.2)	
Complete primary	84.1 (82.2 to 85.7)		75.9 (74.7 to 77.1)	
Incomplete secondary	90.5 (89.7 to 91.2)		79.9 (78.9 to 80.9)	
Complete secondary	89.0 (87.8 to 90.1)		80.3 (78.1 to 82.4)	
Higher	95.4 (94.6 to 96.1)		92.6 (91.0 to 94.0)	
Current working status		<0.001		<0.001
Not working	87.7 (86.8 to 88.6)		60.7 (59.3 to 62.1)	
Working	84.6 (83.7 to 85.5)		66.8 (65.9 to 67.7)	
Marital status		<0.001		<0.001
Never	89.6 (88.3 to 90.7)		77.4 (75.9 to 78.8)	
Married	85.3 (84.3 to 86.3)		61.9 (60.8 to 63.0)	
Cohabiting	85.8 (84.4 to 87.1)		71.6 (70.2 to 73.0)	
Widowed	80.1 (76.1 to 83.6)		60.3 (57.1 to 63.4)	
Divorced	85.7 (82.7 to 88.3)		68.7 (66.2 to 71.0)	
Separated	84.7 (82.6 to 86.6)		67.7 (65.7 to 69.6)	
Parity		<0.001		<0.001
Primiparity	90.8 (90.0 to 91.5)		75.0 (74.0 to 76.0)	
Multiparity	87.0 (86.2 to 87.7)		66.7 (65.7 to 67.7)	
Grandparity	77.1 (75.5 to 78.6)		56.0 (54.8 to 57.1)	
Age at first birth		<0.001		<0.001
Adult	89.9 (89.2 to 90.5)		70.9 (69.9 to 71.8)	
Adolescent	81.9 (80.8 to 82.9)		60.9 (59.9 to 62.0)	
Number of ANC visits		<0.001		<0.001
0	60.7 (57.4 to 63.9)		19.3 (18.0 to 20.6)	
1–3	83.8 (82.6 to 84.9)		64.4 (63.4 to 65.5)	
≥4	89.4 (88.7 to 90.0)		76.9 (76.1 to 77.7)	
Covered by health insurance		<0.001		<0.001
No	84.3 (83.5 to 85.2)		62.7 (61.7 to 63.7)	
Yes	96.2 (95.4 to 96.8)		88.6 (87.4 to 89.8)	
Watches television		<0.001		<0.001
No	76.7 (75.1 to 78.3)		61.2 (60.1 to 62.2)	
Yes	89.6 (88.9 to 90.2)		76.3 (75.2 to 77.4)	
Listens to radio		<0.001		
No	83.4 (82.3 to 84.6)		58.0 (56.9 to 59.2)	
Yes	87.5 (86.7 to 88.2)		72.1 (71.2 to 73.0)	
Reads newspaper or magazine				<0.001
No	83.5 (82.5 to 84.4)		62.8 (61.8 to 63.7)	
Yes	93.4 (92.8 to 94.0)		84.1 (83.1 to 85.1)	
Uses internet		<0.001		<0.001
No	81.2 (80.1 to 82.2)		63.4 (62.5 to 64.4)	
Yes	94.3 (93.7 to 94.8)		87.9 (86.8 to 89.0)	
Getting medical help for self				
Permission to go		0.010		<0.001
Not a big problem	86.2 (85.4 to 87.0)		67.4 (66.5 to 68.4)	
Big problem	84.3 (82.8 to 85.8)		53.2 (51.5 to 54.9)	
Distance to health facility		<0.001		<0.001
Not a big problem	86.8 (86.0 to 87.6)		70.1 (69.0 to 71.1)	
Big problem	83.1 (81.8 to 84.4)		58.5 (57.3 to 59.7)	
Money for treatment		<0.001		<0.001
Not a big problem	87.9 (87.1 to 88.7)		69.8 (68.7 to 70.8)	
Big problem	83.4 (82.3 to 84.4)		61.0 (59.9 to 62.1)	
Not wanting to go alone		0.003		<0.001
Not a big problem	86.2 (85.4 to 87.0)		68.4 (67.5 to 69.4)	
Big problem	84.0 (82.4 to 85.5)		54.4 (53.0 to 55.8)	
Sex of household head		<0.001		<0.001
Male	85.1 (84.2 to 86.0)		63.2 (62.2 to 64.3)	
Female	88.1 (87.1 to 89.0)		70.6 (69.5 to 71.8)	
Wealth index		<0.001		<0.001
Poorest	72.1 (67.9 to 75.9)		53.2 (51.8 to 54.6)	
Poorer	74.6 (71.7 to 77.2)		62.2 (60.9 to 63.4)	
Middle	78.4 (76.7 to 80.0)		70.7 (69.4 to 71.9)	
Richer	86.3 (85.3 to 87.2)		77.3 (75.9 to 78.6)	
Richest	92.7 (92.1 to 93.3)		87.1 (85.5 to 88.5)	

a p-Values obtained from χ^2^ test.

Among rural women, the prevalence of health facility delivery was high among those ages 20–24 years (68.0%), those with higher education (92.6%), those currently working (66.8%), those who have never been married (77.4%), primiparous women (75.0%), those who gave birth as adults (70.9%), those in the richest wealth quintile (87.1%) and those who had four or more ANC visits (76.9%). Also, women who had health insurance (88.6%), used the internet (87.9%), watched television (76.3%), listened to the radio (72.1%) and read newspapers or magazines (84.1%) reported a higher prevalence of health facility delivery. Women who viewed permission to go (67.4%), distance to the health facility (70.1%), getting money for treatment (69.8%) and not wanting to go alone (68.4%) as not a big problem reported higher health facility delivery.

### Factors associated with health facility delivery among women in SSA

#### Urban areas

Compared with women aged 15–19 years, those aged 35–39 years had the highest odds of health facility delivery (aOR 1.93 [95% CI 1.58 to 2.35]). Women with an incomplete primary education (aOR 1.15 [95% CI 1.05 to 1.27]) and those with an incomplete secondary education (aOR 1.35 [95% CI 1.23 to 1.49]) were more likely to have a health facility delivery compared with those with no formal education. However, women with a complete secondary education (aOR 0.82 [95% CI 0.73 to 0.94]) had a lower likelihood of delivering at a health facility than those without education. Separated women were less likely to deliver in health facilities than those who had never married (aOR 0.80 [95% CI 0.66 to 0.97]). For parity, women with multiparity (aOR 0.65 [95% CI 0.58 to 0.73]) and grandparity (aOR 0.39 [95% CI 0.33 to 0.45]) had lower likelihood of health facility delivery relative to those with primiparity. Women whose first pregnancy occurred during adolescence were less likely to deliver in health facilities than those whose first birth occurred during adulthood (aOR 0.78 [95% CI 0.72 to 0.84]). The odds of health facility delivery were high among women who had one to three ANC visits (aOR 3.35 [95% CI 3.01 to 3.72]) and four or more visits (aOR 4.19 [95% CI 3.80 to 4.61]) compared with those with no visit. Women covered by health insurance (aOR 2.83 [95% CI 2.34 to 3.42]), those who read newspapers or magazines (aOR 1.47 [95% CI 1.32 to 1.64]), those who watch television (aOR 1.29 [95% CI 1.20 to 1.39]), those who use the internet (aOR 2.02 [95% CI 1.82 to 2.23]), and those in female-headed households (aOR 1.23 [95% CI 1.12 to 1.34]) were more likely to deliver in health facilities. The odds of health facility delivery increased with increasing wealth index, with the highest odds among women from the richest quintile households (aOR 2.44 [95% CI 2.12 to 2.81]).

#### Rural areas

Women’s age was associated with health facility delivery, with the highest odds among those ages 35–39 years (aOR 1.61 [95% CI 1.46 to 1.78]) compared with those ages 15–19 years. Except for women with complete secondary education, women with incomplete primary (aOR 1.37 [95% CI 1.31 to 1.42]), complete primary (aOR 1.65 [95% CI 1.56 to 1.75]), incomplete secondary (aOR 1.55 [95% CI 1.47 to 1.64]) and higher (aOR 1.77 [95% CI 1.39 to 2.24]) educational levels were more likely to deliver in health facilities. The odds of a health facility delivery was higher among women with one to three ANC visits (aOR 5.87 [95% CI 5.56 to 6.19]), four or more ANC visits (aOR 9.97 [95% CI 9.46 to 10.51]), those covered by health insurance (aOR 2.66 [95% CI 2.45 to 2.89]), those who read newspapers or magazines (aOR 1.42 [95% CI 1.32 to 1.54]), those who listen to the radio (aOR 1.14 [95% CI 1.10 to 1.18]), those who use the internet (aOR 1.59 [95% CI 1.44 to 1.76]), those who watch television (aOR 1.23 [95% CI 1.18 to 1.29]) and those who reside in female-headed households (aOR 1.28 [95% CI 1.22 to 1.34]). Compared with women from the poorest wealth index households, those from the richest households had the highest odds of health facility delivery (aOR 2.45 [95% CI 2.19 to 2.75]). However, parity, age at first birth, distance to the health facility and not wanting to go alone were associated with a lower likelihood of health facility delivery (Table 4).

**Table 4.** Factors associated with health facility delivery among women in SSA.

Variable	Urban, aOR (95% CI)	Rural, aOR (95% CI)
Woman’s age (years)		
15–19	1.00	1.00
20–24	1.13 (0.97 to 1.32)	1.23*** (1.14 to 1.32)
25–29	1.30** (1.09 to 1.54)	1.27*** (1.17 to 1.38)
30–34	1.62*** (1.35 to 1.95)	1.51*** (1.38 to 1.66)
35–39	1.93*** (1.58 to 2.35)	1.61*** (1.46 to 1.78)
40–44	1.72*** (1.38 to 2.14)	1.50*** (1.35 to 1.68)
45–49	1.57** (1.20 to 2.05)	1.36*** (1.19 to 1.56)
Educational attainment		
None	1.00	1.00
Incomplete primary	1.15** (1.05 to 1.27)	1.37*** (1.31 to 1.42)
Complete primary	1.12 (0.99 to 1.27)	1.65*** (1.56 to 1.75)
Incomplete secondary	1.35*** (1.23 to 1.49)	1.55*** (1.47 to 1.64)
Complete secondary	0.82** (0.73 to 0.94)	1.02 (0.91 to 1.15)
Higher	0.96 (0.79 to 1.18)	1.77*** (1.39 to 2.24)
Marital status		
Never	1.00	1.00
Married	1.01 (0.88 to 1.17)	0.83*** (0.77 to 0.90)
Cohabiting	0.99 (0.85 to 1.16)	1.10* (1.01 to 1.20)
Widowed	0.83 (0.63 to 1.10)	0.71*** (0.61 to 0.83)
Divorced	0.92 (0.72 to 1.18)	0.91 (0.79 to 1.04)
Separated	0.80* (0.66 to 0.97)	0.75*** (0.67 to 0.84)
Parity		
Primiparity	1.00	1.00
Multiparity	0.65*** (0.58 to 0.73)	0.64*** (0.60 to 0.68)
Grandparity	0.39*** (0.33 to 0.45)	0.46*** (0.42 to 0.50)
Age at first birth		
Adult	1.00	1.00
Adolescent	0.78*** (0.72 to 0.84)	0.83*** (0.79 to 0.86)
Number of ANC visits		
0	1.00	1.00
1–3	3.35*** (3.01 to 3.72)	5.87*** (5.56 to 6.19)
≥4	4.19*** (3.80 to 4.61)	9.97*** (9.46 to 10.51)
Covered by health insurance		
No	1.00	1.00
Yes	2.83*** (2.34 to 3.42)	2.66*** (2.45 to 2.89)
Reads newspaper or magazine		
No	1.00	1.00
Yes	1.47*** (1.32 to 1.64)	1.42*** (1.32 to 1.54)
Listens to radio		
No	1.00	1.00
Yes	0.91** (0.84 to 0.97)	1.14*** (1.10 to 1.18)
Watches television		
No	1.00	1.00
Yes	1.29*** (1.20 to 1.39)	1.23*** (1.18 to 1.29)
Uses internet		
No	1.00	1.00
Yes	2.02*** (1.82 to 2.23)	1.59*** (1.44 to 1.76)
Getting medical help for self		
Permission to go		
Not a big problem	1.00	1.00
Big problem	1.11* (1.00 to 1.22)	0.98 (0.94 to 1.02)
Distance to health facility		
Not a big problem	1.00	1.00
Big problem	0.90* (0.83 to 0.99)	0.89*** (0.86 to 0.93)
Not wanting to go alone		
Not a big problem	1.00	1.00
Big problem	1.00 (0.90 to 1.12)	0.76*** (0.73 to 0.80)
Sex of household head		
Male	1.00	1.00
Female	1.23*** (1.12 to 1.34)	1.28*** (1.22 to 1.34)
Wealth index		
Poorest	1.00	1.00
Poorer	0.93 (0.81 to 1.07)	1.22*** (1.18 to 1.27)
Middle	1.11 (0.98 to 1.26)	1.52*** (1.46 to 1.59)
Richer	1.71*** (1.50 to 1.95)	1.77*** (1.66 to 1.87)
Richest	2.44*** (2.12 to 2.81)	2.45*** (2.19 to 2.75)
*N*	56 596	116 197
Pseudo-*R*^2^	0.143	0.184

Exponentiated coefficients. *p<0.05, **p<0.01, ***p<0.001.

### Decomposition analysis of factors contributing to inequalities in health facility delivery

Table 5 presents the results from the decomposition analysis of the factors contributing to rural–urban disparities. The results indicate that 87.13% of the observed rural–urban disparity in health facility delivery was explained by differences in women’s characteristics. These estimates reflect pooled averages across the 23 sub-Saharan African countries rather than individual country-level results. Being in the richest wealth quintile and having four or more ANC visits were the characteristics that explained most, 15.62% and 10.55%, respectively, to the rural–urban disparities in health facility delivery. Other notable contributing characteristics were use of the internet (4.72%) and having no formal education (4.43%).

**Table 5.** Decomposition analysis of factors contributing to inequalities in health facility delivery.

	Difference due to characteristics (E)		Difference due to coefficients (C)	
Variable	Coefficient	%	Coefficient	%
% total explained disparity	−0.173 61***	87.13	−0.025 63***	12.87
Woman’s age (years)				
15–19	−0.000 68***	0.34	−0.000 06	0.03
20–24	−0.000 14***	0.07	0.004 09**	−2.05
25–29	0.000 21**	−0.10	0.002 21	−1.11
30–34	−0.000 33***	0.16	0.000 73	−0.37
35–39	−0.000 05***	0.01	−0.001 76*	0.88
40–44	0.000 21***	−0.11	−0.000 52	0.26
45–49	0.000 07	−0.03	−0.000 18	0.09
Educational attainment				
None	−0.008 83***	4.43	−0.005 52***	2.77
Incomplete primary	0.001 04***	−0.52	−0.000 79	0.40
Complete primary	0.000 33***	−0.16	0.001 89**	−0.95
Incomplete secondary	−0.003 98***	2.00	−0.002 05	1.03
Complete secondary	0.004 13***	−2.07	−0.001 22	0.61
Higher	−0.002 03*	1.02	0.002 16	−1.08
Marital status				
Never	−0.000 17	0.09	−0.000 04	0.02
Married	0.000 02	−0.01	−0.008 21*	4.12
Cohabiting	−0.000 36***	0.18	0.002 26*	−1.14
Widowed	−0.000 01**	0.00	−0.000 13	0.07
Divorced	−0.000 06**	0.03	0.000 39	−0.20
Separated	0.000 08***	−0.04	−0.000 25	0.13
Parity				
Primiparity	−0.003 91***	1.96	−0.000 46	0.23
Multiparity	0.000 52***	−0.26	−0.003 95*	1.98
Grandparity	−0.006 65***	3.34	0.002 09*	−1.05
Age at first birth				
Adult	−0.001 50***	0.75	−0.002 35*	1.18
Adolescent	−0.001 50***	0.75	0.002 43*	−1.22
Number of ANC visits				
0	−0.013 09***	6.57	−0.003 18***	1.59
1–3	0.005 38***	−2.70	−0.000 09	0.04
≥4	−0.021 02***	10.55	0.027 14***	−13.62
Covered by health insurance				
No	−0.003 06***	1.54	−0.006 89	3.46
Yes	−0.003 06***	1.54	0.000 87	−0.44
Reads newspaper or magazine				
No	−0.003 44***	1.73	−0.005 09	2.56
Yes	−0.003 44***	1.73	0.001 42	−0.71
Listens to radio				
No	−0.001 23***	0.62	−0.003 55***	1.78
Yes	−0.001 23***	0.62	0.005 68***	−2.85
Watches television				
No	−0.004 84***	2.43	−0.000 48	0.24
Yes	−0.004 84***	2.43	0.000 99	−0.50
Uses internet				
No	−0.009 40***	4.72	0.008 06**	−4.04
Yes	−0.009 40***	4.72	−0.003 53**	1.77
Getting medical help for self				
Permission to go				
Not a big problem	0.000 01	−0.01	0.003 64	−1.83
Big problem	0.000 01	−0.01	−0.000 70	0.35
Distance to health facility				
Not a big problem	−0.002 28***	1.14	−0.001 78	0.89
Big problem	−0.002 28***	1.14	0.000 55	−0.28
Not wanting to go alone				
Not a big problem	−0.002 47***	1.24	0.009 58	−4.81
Big problem	−0.002 47***	1.24	−0.001 67	0.84
Sex of household head				
Male	−0.000 94***	0.47	−0.003 96*	1.99
Female	−0.000 94***	0.47	0.001 40*	−0.70
Wealth index				
Poorest	−0.021 20***	10.64	−0.000 94**	0.47
Poorer	−0.008 42***	4.22	0.002 43***	−1.22
Middle	−0.000 04	0.02	0.004 29***	−2.15
Richer	−0.005 22***	2.62	−0.003 87**	1.94
Richest	−0.031 12***	15.62	−0.008 85***	4.44

Exponentiated coefficients. *p<0.05, **p<0.01, ***p<0.001.

## Discussion

This study examined rural–urban disparities in health facility delivery among women in 23 sub-Saharan African countries. We found significant differences, with nearly three-quarters of women (72.1%) delivering in a health facility. Key factors associated with facility delivery included increased age, ANC visits, educational attainment, health insurance coverage, sex of household head, wealth index and exposure to media (newspapers, television and the internet). Decomposition analysis revealed that wealth index, ANC visits, internet use and level of education were the major factors that explained rural–urban disparities in health facility delivery. Given that the analysis pooled data from 23 countries with diverse health systems, policy environments and sociocultural contexts, these findings should be interpreted as reflecting regional patterns rather than being uniformly applicable country-level effects. The findings should be considered alongside evidence from individual countries to develop policies and interventions tailored to specific contexts.

The estimated prevalence in this study is higher than the 66% reported by Adde et al.^31^; however, it remains lower than the 82.3% prevalence reported by Gebremichael and Fenta,^35^ possibly due to differences in study populations, time frames, improvements in maternal health services over time, healthcare infrastructure, policies promoting health facility deliveries or regional disparities in healthcare access.^36–38^ Our study found that the proportion of deliveries in health facilities was significantly higher in urban areas (85.9%) than in rural areas (64.7%). This disparity aligns with previous research in East Africa^39^ and other LMICs.^20,28,40–42^ This demonstrates the persistent urban advantage in maternal healthcare access.

Urban areas typically have better healthcare infrastructure, shorter travel distances to health facilities and greater exposure to health information, all of which contribute to higher health facility delivery rates.^43^ Factors consistently associated with health facility deliveries include maternal education, household wealth, urban residence, proximity to health facilities and number of ANC visits.^44^ In the urban slums of Kampala, Uganda, 66.1% of women delivered in health facilities, with media exposure, the timing of the first ANC visit and the number of ANC visits being significant predictors.^45^ Healthcare facilities in rural areas of SSA are often in poor condition, which may limit the use of maternal healthcare services.^46^ Additionally, the lack of incentives for qualified healthcare workers discourages them from serving in rural communities.^47,48^ Distance to health facilities remains a crucial factor, with each additional kilometre decreasing the odds of institutional delivery by 3% in Pakistan.^49^ However, even in urban areas with better physical access, a significant proportion of women still do not deliver in health facilities, suggesting that other factors, such as service quality and awareness, also play important roles.^43,45^

Consistent with previous studies, the odds of giving birth at a health facility were higher among women with increasing age, more ANC visits, media exposure and greater wealth, education and health insurance, regardless of their place of residence.^19,50,51^ The strong association with ANC for both rural and urban dwellers reflects its dual role, not only as an individual-level predictor of maternal health-seeking behaviour but also as a vital service delivery platform that connects women to essential maternal health services, promotes health education and facilitates birth preparedness. Women from wealthier households in both rural and urban areas had greater odds of delivering in a health facility.^19,50^ Conversely, multiparity, adolescent first births and perceiving distance as a barrier were linked to reduced odds of facility delivery.^50,51^

In urban areas, higher education was associated with an increased likelihood of health facility delivery.^50^ This pattern should be interpreted in context. In urban settings, where health facilities are more accessible and services are better resourced, education may more readily translate into increased health literacy, autonomy and effective use of maternal health services. In contrast, in rural areas, the influence of education on health facility delivery may be constrained by persistent structural barriers such as long distances to facilities, transportation challenges and limited service availability. Importantly, this does not imply that education is unimportant in rural settings; rather, the observed differences highlight that structural and access-related barriers in rural areas may limit women’s ability to translate educational attainment into greater use of health facilities.

National health insurance and free maternal healthcare policies in SSA have increased the number of deliveries at health facilities and improved maternal health outcomes. For instance, evidence from Ghana suggests that implementing national health insurance led to a significant increase in deliveries at health facilities, particularly among the poorest populations.^52^ This highlights the importance of financial protection in reducing economic barriers to maternal healthcare. Expanding and strengthening such policies across SSA, especially in rural areas where affordability remains a key challenge, could help bridge the observed rural–urban disparities in health facility deliveries.

We found that women in female-headed households are more likely to utilise health facility delivery services compared with those in male-headed households, which can be attributed to gender norms and decision-making dynamics. Research indicates that male involvement in maternal healthcare can significantly improve outcomes in LMICs. Studies have shown that engaging men in ANC is associated with increased utilisation of skilled birth attendance, institutional delivery and postpartum care.^53^ Women’s empowerment in decision-making and employment positively influences maternal care utilisation.^54^ These findings suggest that targeting gender-based barriers through male involvement is crucial for improving facility delivery rates.

The decomposition analysis results indicate that being in the richest wealth quintile, having four or more ANC visits and having no formal education were the key factors contributing to rural–urban disparities in health facility delivery. These findings highlight critical socio-economic and information barriers driving these disparities. The strong influence of wealth status underscores the need for financial interventions to help rural and low-income women access skilled delivery services. This aligns with previous studies in SSA, which show that rural women face greater challenges in accessing maternal healthcare services.^19,20,48^ Increasing ANC coverage and addressing transportation difficulties, particularly in rural areas, could also help bridge the gap, as more ANC visits are associated with higher health facility delivery rates.^50^ To reduce maternal health inequalities, interventions should focus on socio-economic empowerment, targeted awareness campaigns and improving access to health facilities in rural areas.^19,20^ Additionally, promoting digital health education and expanding internet access may improve maternal health awareness, while targeted educational campaigns for women with low literacy levels could further encourage deliveries at health facilities.^55^

Studies in rural regions highlight that women often prefer home births over institutional deliveries due to several barriers. Distance to health facilities, transportation costs and geographical challenges are significant obstacles to health services.^56,57^ Cultural preferences for traditional birth practices and family involvement also influence this decision.^56,58^ Additionally, perceived poor quality of care and negative attitudes of skilled birth attendants deter many women from seeking institutional deliveries.^57^ Financial constraints and the influence of family members, especially mothers-in-law, further contribute to the preference for home births.^58,59^ These factors indicate the multifaceted nature of the barriers women face in accessing health facility deliveries; therefore, further research is necessary to comprehensively address these barriers and improve maternal health outcomes, particularly in rural settings.

### Policy implications

While these findings provide a regional overview of residence-based inequalities in health facility delivery across SSA, policy responses should be tailored to country-specific health system contexts and informed by national and subnational evidence. Our findings highlight significant pro-urban disparities in health facility delivery in SSA, underscoring the need for targeted interventions to address these gaps in line with global maternal health strategies, such as the WHO’s Ending Preventable Maternal Mortality (EPMM) strategy, which aims to reduce global maternal mortality to 70 per 100 000 live births by 2030.^60^ The EPMM strategy focuses on equitable access to quality maternal healthcare, aligning with our findings that wealth and education play pivotal roles in determining the delivery of healthcare at health facilities. While upstream determinants such as household wealth and maternal education strongly drive rural–urban disparities, these factors are slow to change and require long-term socio-economic interventions. In contrast, health system levers such as ensuring continuity of ANC, implementing community outreach programs, providing counselling, strengthening referral systems and improving transport support are more immediately actionable. Policymakers can prioritise these interventions to reduce rural–urban gaps in health facility delivery in the short term, while longer-term strategies focus on addressing structural inequalities. As such, long-term interventions are needed to address upstream determinants; public health stakeholders in SSA should prioritise initiatives that empower women economically, improve household income, and help reduce wealth-based disparities in maternal health.^61^

Additionally, the WHO’s Global Strategy for Women’s, Children’s, and Adolescents’ Health^62^ emphasises the importance of ANC in improving maternal health outcomes. Strengthening ANC advocacy and ensuring access to timely, high-quality ANC services is crucial to reducing disparities in health facility delivery. Health departments across SSA must increase efforts to encourage regular ANC visits, especially in underserved areas. Furthermore, our findings suggest the importance of involving male partners in decisions related to maternal health. This aligns with global initiatives such as the global framework for assessing male involvement in maternal health,^63^ which advocates for the inclusion of men in maternal health programs to improve outcomes for women and children.

Countries such as Ethiopia, Rwanda, Tanzania and Ghana provide illustrative examples of how context-appropriate health system strategies can improve access to maternal healthcare and increase health facility deliveries rather than representing exhaustive or country-specific policy evaluations. Ghana’s free maternal healthcare policy and CHPS program have demonstrated improvement in access to skilled delivery care, particularly among underserved populations.^64,65^ Similarly, Rwanda’s community-based health insurance and Tanzania’s community health worker network highlight the role of financial protection and community-level service delivery in reducing geographic and socio-economic barriers to maternal healthcare.^66,67^ Collectively, these experiences underscore the importance of integrated, equity-focused health system approaches that can be adapted across SSA, consistent with the regional patterns identified in this study.

### Strengths and limitations

The utilisation of decomposition analysis provided valuable insights into the determinants of socio-economic disparities. Furthermore, the large sample size allows us to generalise the findings to the broader population of women of reproductive age in SSA. However, there are notable limitations to consider. Causal relationships among factors linked to rural–urban disparities in health facility delivery cannot be established, as the dataset is cross-sectional. Additionally, the explanatory variables available in the DHS primarily capture demand-side and household-level characteristics. Important supply-side aspects of care, such as health facility readiness, availability of skilled staff, quality of care, referral systems and transport infrastructure, are not comprehensively measured in the DHS and cannot be included in the analysis. Consequently, the findings should be interpreted as reflecting compositional and demand-side drivers of rural–urban inequalities rather than a complete assessment of health system performance. Decomposition analysis assumes a linear, additive relationship between socio-economic factors and healthcare outcomes, which may not always be applicable. Women’s decisions regarding health facility delivery are also influenced by broader sociocultural factors such as cultural and religious beliefs and partner approval, which are beyond the scope of this study.

## Conclusions

This study highlights significant disparities in health facility delivery utilisation in SSA, particularly the rural–urban divide. Our decomposition analysis reveals that wealth index, ANC visits, education and internet use contribute to disparities in maternal healthcare access. To address these disparities, targeted interventions are needed. Economic empowerment programs should focus on improving women’s income, particularly in rural areas, through microcredit and livelihood initiatives. Educational campaigns can enhance women’s understanding of the benefits of health facility deliveries and increase their earning potential. Strengthening ANC services and promoting early and consistent ANC visits, especially in underserved areas, is essential. Furthermore, male involvement programs should be prioritised to encourage shared decision-making and support for health facility delivery. With a focus on these areas, policies can improve maternal health outcomes and help achieve SDG 3, aiming to reduce maternal mortality to <70 deaths per 100 000 live births by 2030.

## Data Availability

The data used for this study are freely available at http://dhsprogram.com/data/available-datasets.cfm.
